# CRKL promotes hepatocarcinoma through enhancing glucose metabolism of cancer cells via activating PI3K/Akt

**DOI:** 10.1111/jcmm.16303

**Published:** 2021-02-01

**Authors:** Chunmei Guo, Chao Gao, Xinxin Lv, Dongting Zhao, Frederick T. Greenaway, Lihong Hao, Yuxiang Tian, Shuqing Liu, Ming‐Zhong Sun

**Affiliations:** ^1^ Department of Biotechnology College of Basic Medical Sciences Dalian Medical University Dalian China; ^2^ Carlson School of Chemistry and Biochemistry Clark University Worcester MA USA; ^3^ Department of Histology and Embryology College of Basic Medical Sciences Dalian Medical University Dalian China; ^4^ Department of Biochemistry College of Basic Medical Sciences Dalian Medical University Dalian China; ^5^ Present address: College of Medical Laboratory Science and Technology, Harbin Medical University (Daqing) Daqing China

**Keywords:** CRKL, glucose metabolism, hepatocarcinoma, PI3K/Akt pathway

## Abstract

Abnormal glucose metabolism may contribute to cancer progression. As a member of the CRK (v‐crk sarcoma virus CT10 oncogene homologue) adapter protein family, CRKL (CRK‐like) associated with the development and progression of various tumours. However, the exact role and underlying mechanism of CRKL on energy metabolism remain unknown. In this study, we investigated the effect of CRKL on glucose metabolism of hepatocarcinoma cells. CRKL and PI3K were found to be overexpressed in both hepatocarcinoma cells and tissues; meanwhile, CRKL up‐regulation was positively correlated with PI3K up‐regulation. Functional investigations revealed that CRKL overexpression promoted glucose uptake, lactate production and glycogen synthesis of hepatocarcinoma cells by up‐regulating glucose transporters 1 (GLUT1), hexokinase II (HKII) expression and down‐regulating glycogen synthase kinase 3β (GSK3β) expression. Mechanistically, CRKL promoted glucose metabolism of hepatocarcinoma cells via enhancing the CRKL‐PI3K/Akt‐GLUT1/HKII‐glucose uptake, CRKL‐PI3K/Akt‐HKII‐glucose‐lactate production and CRKL‐PI3K/Akt‐Gsk3β‐glycogen synthesis. We demonstrate CRKL facilitates HCC malignancy via enhancing glucose uptake, lactate production and glycogen synthesis through PI3K/Akt pathway. It provides interesting fundamental clues to CRKL‐related carcinogenesis through glucose metabolism and offers novel therapeutic strategies for hepatocarcinoma.

## 1 INTRODUCTION

Hepatocarcinoma (HCC) is the most common primary neoplasm of liver with the highest morbidity and mortality rate in the world.^1^, ^2^ Marked progress in hepatocarcinoma treatment has been achieved by the combination of surgical resection and chemotherapy.^3^ However, the relative higher metastasis, recurrence and chemoresistance of hepatocarcinoma still leads to poorer prognosis of patients.^4^ Therefore, improvement in the diagnosis and treatment of hepatocarcinoma depends on boosting our comprehending for the underlying molecular mechanisms controlling its development and progression. Identifying potential indicators for the progression and drug‐tolerance of hepatocarcinoma will lead to better diagnosis and treatment of the patients.

Emerging as a novel hallmark of cancer, energy metabolism reprogramming is crucial for the proliferation, differentiation and metastasis of cells.^5^ Even in the presence of enough oxygen, tumour cells enhance glucose consumption with increased lactate production through aerobic glycolysis instead of oxidative phosphorylation for their growths.^6^ Cancer cells preferentially selected aerobic glycolysis over oxidative phosphorylation for glucose‐dependent ATP production due to mitochondrial impairments.^7^, ^8^ Increased glycolysis benefits tumour cell growth and dissemination. Lactate accumulation results in an acidic extracellular microenvironment causing the death of surrounding normal cells, ECM remodelling, promoting EMT and cancer cells metastasis.^9^, ^10^, ^11^ Uncovering the mechanisms underlying the Warburg effect is helpful for the development of novel therapeutic targets and strategies of human cancers.

**FIGURE 1:**
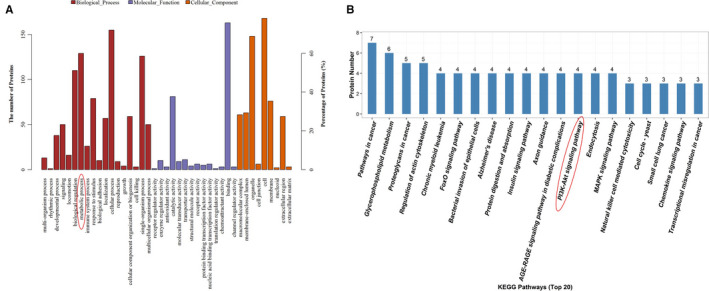
iTRAQ quantitative proteomic screening the differentially expressed proteins in K562 cells with CRKL knockdown. A, GO analysis of the differentially expressed proteins involved in cellular processes. B, KEGG analysis of the differentially expressed proteins involved in signal transduction pathway

As a member of the CRK (v‐crk sarcoma virus CT10 oncogene homologue) adapter protein family, CRKL (CRK‐like) is ubiquitously expressed in eukaryotic organisms.^12^ CRKL has various linkages for combining to BCR‐ABL, GAB, C3G, Pax, GEF and SOS to form complexes that are crucial for cell survival, proliferation, migration and adhesion.^13^, ^14^, ^15^ Hence, CRKL can function in cell signalling pathway by either directly forming complex with downstream receptor molecules to mediate tyrosine kinase activity, or by acting as an upstream regulator for signal initiation.^15^, ^16^ The deregulation of CRKL associated with the development and progression of cancers.^17^, ^18^ We previously found that CRKL deregulation was remarkably affecting the metastasis capacities of HCC cells.^19^, ^20^ However, the biological function and regulatory mechanism of CRKL in metabolism are unknown. Previously, we screened the differentially expressed proteins in K562 cells with CRKL knockdown by iTRAQ quantitative proteomics, Gene ontology (GO) analysis revealed that CRKL deregulation related to metabolic process (Figure 1A). We speculate that CRKL may affect glucose metabolism of hepatocarcinoma cells.

In the present study, we report a novel biological function of CRKL in glucose metabolism of hepatocarcinoma. We found that CRKL and PI3K were up‐regulated in HCC cells and tissues. Moreover, CRKL overexpression facilitated the Warburg effect and glycogen synthesis of hepatocarcinoma cells. Further investigation revealed that CRKL promoted glucose metabolism of hepatocarcinoma cells by activating the PI3K/AKT pathway. Our study has uncovered a novel CRKL‐PI3K/AKT regulatory pathway in glucose metabolism of hepatocarcinoma and partially elucidated the molecular mechanism underlying glucose metabolism in hepatocarcinoma cells.

## 2 MATERIALS AND METHODS

### 2.1 Patient samples

Ten pairs of matched HCC patient tumour tissues and corresponding normal liver tissues were acquired from the Second Affiliated Hospital of Dalian Medical University. There are 8 male patients and 2 female patients, 5 patients ≥ 60 years and 5 patients < 60 years; 5 patients are primary HCC and 5 patients unknown; 3, 5 and 1 patients are T1, T2 and T3 stages, 1 patient unknown. The research approach was approved by the Ethical Committee of Dalian Medical University with approval number 2019‐014, and informed consent was signed by all patients.

### 2.2 Cell culture

Human hepatocarcinoma HepG2, HuH7, HCCLM3 and normal liver LO2 cells were obtained from Cell Bank of the Type Culture Collection, Shanghai Institute of Cell Biology, Chinese Academy of Sciences. All cells were cultured in DMEM (Gibco) medium with 10% foetal bovine serum (FBS, TransGen) at 37°C in a humidified atmosphere with 5% CO_2_. HCCLM3‐PCDH, HCCLM3‐PCDH‐CRKL, HuH7‐PCDH and HuH7‐PCDH‐CRKL cells, which our group previously constructed,^20^ were also grown in 10% FBS supplemented with DMEM.

### 2.3 Western blotting (WB) assay

Protein was extracted from cells and tissues using ice‐cold RIPA buffer as previously described.^20^ Equivalent amounts of protein determined by Bradford method and separated by 10% SDS‐PAGE. The protein bands were electrotransferred to nitrocellulose (NC) membrane (PALL), blocked with 5% skim milk (BD) and subsequently incubated with the primary antibodies CRKL (1:2000, Santa Cruz Biotechnology), PI3K (1:500, Sanying), p‐Akt (1:800, pThr308/Ser473, Cell Signaling), GLUT1 (Glucose transporters1, 1:500, Wanlei Biotechnology), HKII (Hexokinase II, 1:500, Wanlei Biotechnology), GSK3β (Glycogen syntheses kinase 3β, 1:500, Wanlei Biotechnology), ACTB (1:5000, TransGen Biotech) and GAPDH (1:5000, Sanying) at 4°C overnight. After incubation with the secondary antibody at RT for 3 hours, protein bands were observed by ECL and quantified using the Bio‐Rad ChemiDoc™ MP system (Bio‐Rad).

### 2.4 Co‐immunoprecipitation (Co‐IP) assay

The interaction between CRKL and PI3K was determined by Co‐IP assay as previously described.^20^ Protein was extracted from 5 × 10^6^ HCCLM3, HCCLM3‐PCDH or HCCLM3‐PCDH‐CRKL cells by RIPA buffer; then, protein was incubated with control anti‐rabbit IgG (Santa Cruz Biotechnology) or anti‐CRKL (Santa Cruz Biotechnology), PI3K (Sanying) for 1 hour at 4°C. The immune complexes were pulled down by protein A/G magnetic beads (Santa Cruz Biotechnology) overnight at 4°C. After washing with RIPA buffer for 3 times, the immunoprecipitated proteins were detected by WB.

### 2.5 Glucose oxidase‐peroxidase (GOD‐POD) assay

The effect of CRKL up‐regulation on glucose uptake of hepatocarcinoma cells was detected by glucose detection kit (Nanjing Jiancheng Bioengineering Institute). Each group 1 × 10^4^ cells in 200 μL 10% FBS supplemented with DMEM from each group were seeded into a separate well of 96‐well plate, incubated at 37°C with 5% CO_2_ for 24 hours, removed into a 0.6 mL Eppendorf tube and centrifugated with 560 *g* for 10 minutes. Then, 3 μL of supernatant from each group was mixed well with 300 μL of working solution already loaded in a 96‐well pate and incubated at 37°C for 15 minutes. The absorbances at 505 nm were measured using a microplate reader (Thermo). Meanwhile, 3 μL of calibrator (5.55 mmol/L) was used for calibration. Results were the averages from triplicate measurements.

### 2.6 Lactate oxidase (LOD) assay

The effect of CRKL overexpression on lactate production of hepatocarcinoma cells was measured using a lactate detection kit (Nanjing Jiancheng Bioengineering Institute). Each group 1 × 10^4^ cells in 200 μL 10% FBS supplemented with DMEM from each group were seeded into a separate well of 96‐well plate, incubated at 37°C with 5% CO_2_ for 6, 12, 18 and 24 hours, removed into a 0.6 mL Eppendorf tube and centrifugated with 560 *g* for 10 minutes. Then, 2 μL of supernatant from each group was mixed well with 100 μL enzyme working liquid and 20 μL chromogenic reagent in a 96‐well pate, and incubated at 37°C for 10 minutes. Then, 200 μL stop reagent was added to each well for absorbance assay at 530 nm using a microplate reader (Thermo). Meanwhile, 2 μL of calibrator (3 mmol/L) was used for calibration. Results were the averages from triplicate measurements.

### 2.7 Periodic acid Schiff reaction (PAS) assay

The effect of CRKL overexpression on glycogen synthesis of hepatocarcinoma cells was measured by a glycogen staining kit (Wanlei Biotechnology). The 25 mm diameter cover glasses were placed into 6‐well plates, 4 × 10^4^ cells in 100 μL 10% FBS supplemented with DMEM from each group were loaded into a well of 6‐well plate with a 25 mm cover glass and incubated at 37°C for 24 hours. Then, the cover glass was washed with PBS, fixed with ice‐cold acetone, incubated with periodic acid and washed with distilled water. Then, the cover glasses were incubated in 100 μL Schiff for 15 minutes, stained with haematoxylin, clarified by xylene, dehydrated by gradient ethanol and imaged by an upright microscope (Olympus) with 400× and 1600×. Amaranth spherical particle in cytoplasm refers to the PAS‐positive reaction.

### 2.8 Statistical analysis

All data analysis was completed using GraphPad Prism 5.0 software (GraphPad Software Inc). Student's *t* test was used for comparison between two groups. The correlation between the expression level of CRKL and PI3K was analysed by the Spearman's rank correlation coefficient. *P* ≤ .05 was considered statistically significant. All experiments were performed in triplicate for quantitative comparison.

## 3 RESULTS

### 3.1 The expression patterns and correlation of CRKL and PI3K in HCC tissues and cells

We found CRKL expression was up‐regulated in HCC tumour tissues by IHC and WB assays.^19^, ^20^ Consistently, CRKL was more abundant in HepG2, HCCLM3 and HuH7 cells compared with normal liver LO2 cells.^20^ These showed the important role of CRKL in hepatocarcinogenesis.

**FIGURE 2:**
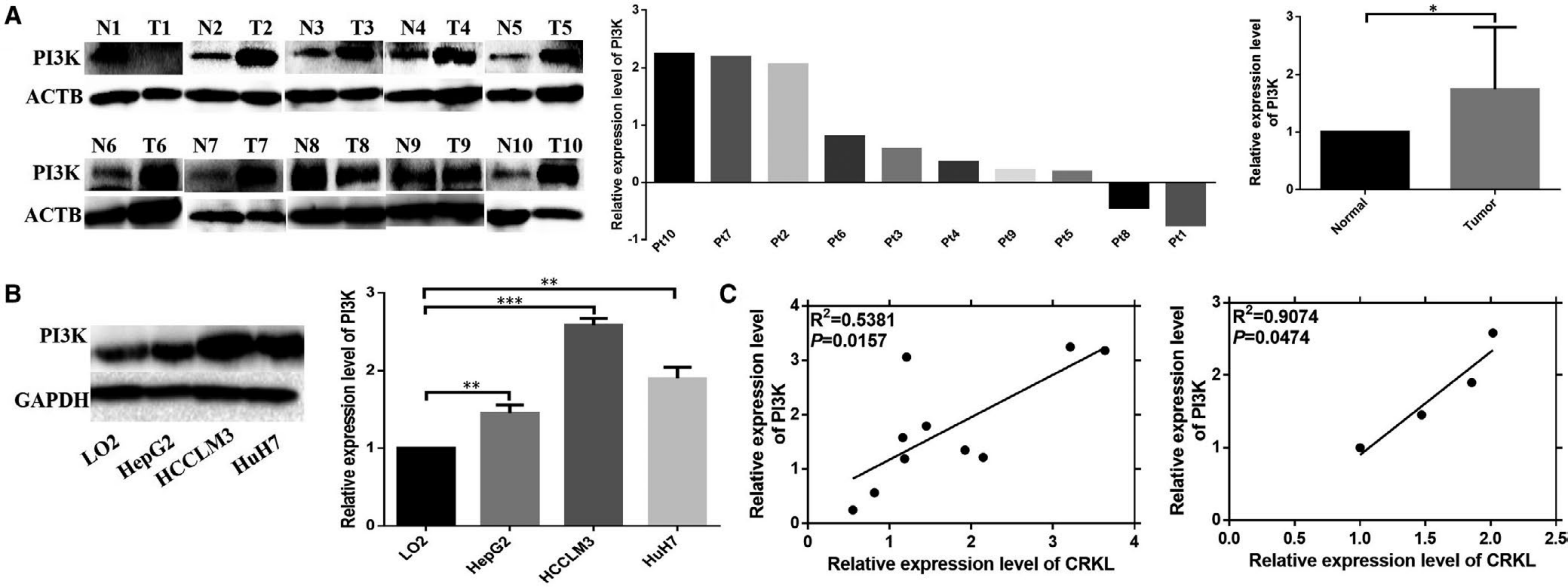
The different expressions and correlationship of PI3K and CRKL in HCC tissues and cells. WB assays indicated that PI3K was overexpressed in HCC tumourous tissues compared with paracancerous normal liver tissues (A) and in HCC HepG2, HCCLM3 and HuH7 cell lines compared with normal liver LO2 cells (B). C, The expression level of CRKL was positively correlated with PI3K expression in both HCC tissues and cells

We detected PI3K was overexpressed in HCC tissues and cells. WB assay showed that PI3K expression was up‐regulated by 74.5% (*P* = .0422) in the tumour tissues from 10 HCC patients (Figure 2A). Meanwhile, PI3K was expressed in all the tested cells, and a comparatively higher expression was exhibited in hepatocarcinoma cells compared to normal liver LO2 cells. The protein expression levels of PI3K were increased by 45.4% (*P* = .0016), 158.3% (*P* = .0005) and 90.0% (*P* = .0040) in HepG2, HCCLM3 and HuH7 compared to LO2 cells (Figure 2B). The results demonstrated the involvement of PI3K overexpression in hepatocarcinogenesis. Taken together, our results indicate that PI3K may act as a tumour promoter involved in the development and progression of HCC.

We further analysed the inter‐correlation of CRKL and PI3K expression. The up‐regulations of CRKL and PI3K were positively correlated in both HCC patients’ tissues (Figure 2C, *R* ^2^ = .5381, *P* = .0157) and cells (Figure 2C, *R* ^2^ = .9074, *P* = .0474). Our results demonstrate that the expression of CRKL is positively related with PI3K, and the dysexpressions of CRKL and PI3K are closely correlated in affecting hepatocarcinoma malignancy.

### 3.2 CRKL promotes the Warburg effect of hepatocarcinoma cells

**FIGURE 3:**
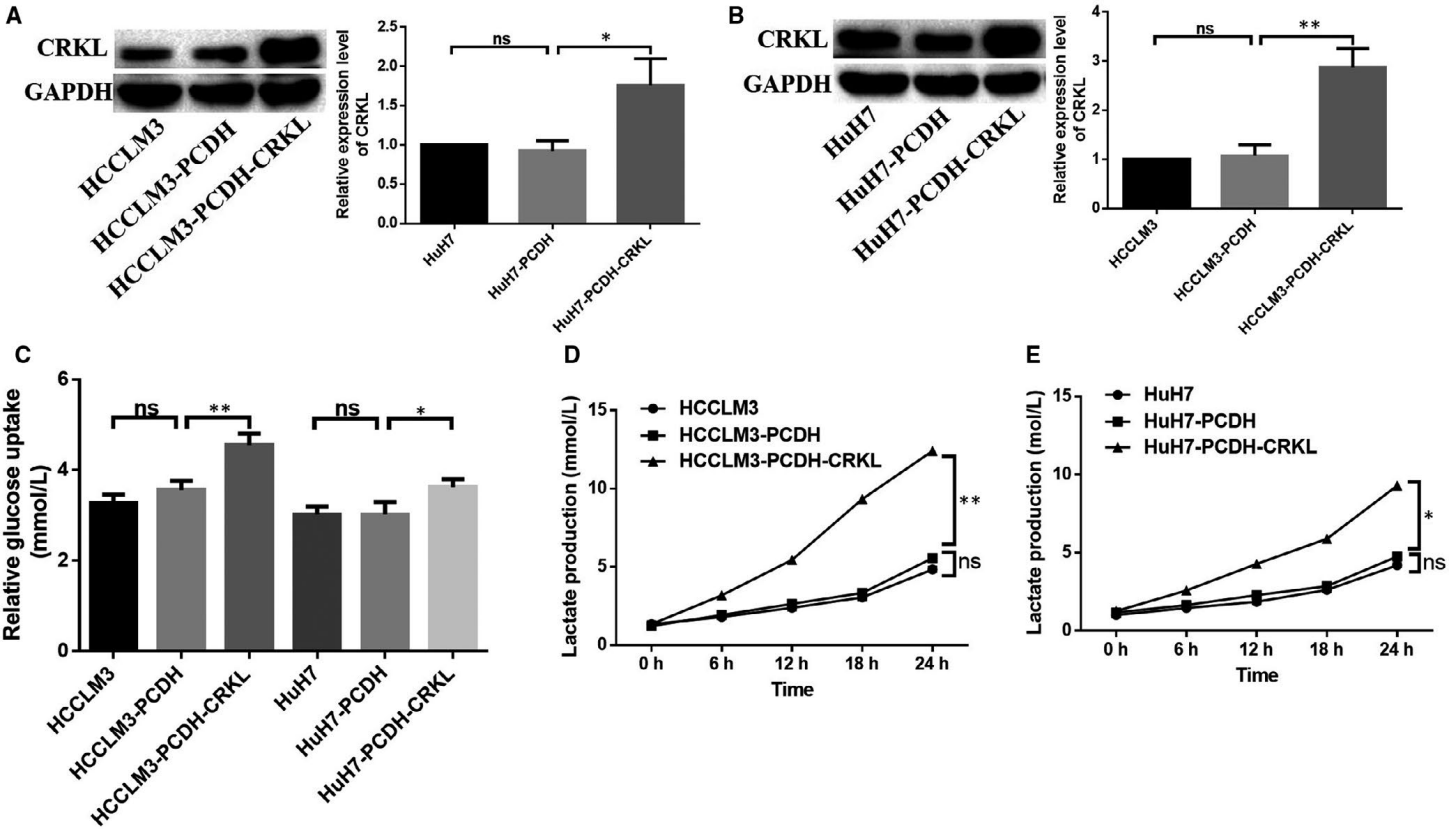
CRKL promotes Warburg effect of hepatocarcinoma cells. A and B, CRKL were stably overexpressed in HCCLM3 and HuH7 cells. C, CRKL enhanced glucose uptake of hepatocarcinoma cells by GOD‐POD assay. D and E, CRKL enhanced lactate production of hepatocarcinoma cells by LOD assay

Warburg effect is a hallmark of cancer cell growth and metastasis. Previously, our group showed the stable CRKL overexpressions in HCCLM3 and HuH7 cells, HCCLM3‐PCDH‐CRKL and HuH7‐PCDH‐CRKL, leading to their invasive behaviours.^20^ CRKL protein levels were increased by 179.3% (*P* = .0023, Figure 3A) and 83.7% (*P* = .0162, Figure 3B) in HCCLM3‐PCDH‐CRKL and HuH7‐PCDH‐CRKL cells compared with HCCLM3‐PCDH and HuH7‐PCDH cells, respectively, while there were no CRKL protein expression level differences between HCCLM3 and HCCLM3‐PCDH (Figure 3A), or HuH7 and HuH7‐PCDH cells (Figure 3B). Herein, we found CRKL up‐regulation promoted glucose metabolism of HCCLM3 and HuH7 cells.

GOD‐POD assay showed CRKL enhanced glucose uptake of hepatocarcinoma cells (Figure 3C, Table 1). No glucose uptake differences were observed between HCCLM3 and HCCLM3‐PCDH (Figure 3C), or in HuH7 and HuH7‐PCDH cells (Figure 3C). In comparison with HCCLM3‐PCDH and HuH7‐PCDH cells, the glucose uptake was increased by 27.8% (*P* = .0065, Figure 3C) and 21.9% (*P* = .0313, Figure 3C) in HCCLM3‐PCDH‐CRKL and HuH7‐PCDH‐CRKL cells, respectively.

**TABLE 1:** The relative glucose uptake amounts of hepatocarcinoma cells

Cell	Glucose uptake (mmol/L)	Cell	Glucose uptake (mmol/L)
HCCLM3	3.309 ± 0.057	HuH7	2.987 ± 0.034
HCCLM3‐PCDH	3.561 ± 0.112	HuH7‐PCDH	3.085 ± 0.065
HCCLM3‐PCDH‐CRKL	4.552 ± 0.156	HuH7‐PCDH‐CRKL	3.761 ± 0.044

LOD assay showed CRKL enhanced lactate production of hepatocarcinoma cells (Figure 3D,E, Table 2). No lactate production differences were observed between HCCLM3 and HCCLM3‐PCDH (Figure 3D), or in HuH7 and HuH7‐PCDH cells (Figure 3E). In comparison with HCCLM3‐PCDH or HuH7‐PCDH, the lactate production was increased by 65.4% (*P* = .0156), 107.1% (*P* = .0132), 212.4% (*P* = .0009), 160.7% (*P* = .0013) or 57.4% (*P* = .0340), 87.5% (*P* = .0142), 106.0% (*P* = .0013), 95.2% (*P* = .0140) in HCCLM3‐PCDH‐CRKL (Figure 3D) or HuH7‐PCDH cells (Figure 3E) at the inoculation time intervals of 6, 12, 18 and 24 hours, respectively. Taken together, the above findings strongly support the notion that CRKL enhances the Warburg effect of hepatocarcinoma cells.

**TABLE 2:** The lactate production amounts of hepatocarcinoma cells

Cell	0 h (mmol/L)	6 h (mmol/L)	12 h (mmol/L)	18 h (mmol/L)	24 h (mmol/L)
HCCLM3	1.330 ± 0.063	1.796 ± 0.397	2.378 ± 0.052	3.050 ± 0.066	4.834 ± 0.052
HCCLM3‐PCDH	1.222 ± 0.247	1.922 ± 0.235	2.630 ± 0.298	3.330 ± 0.270	5.560 ± 0.175
HCCLM3‐PCDH‐CRKL	1.328 ± 0.193	3.178 ± 0.315	5.448 ± 0.060	10.340 ± 0.075	12.41 ± 0.389
HuH7	1.006 ± 0.041	1.452 ± 0.228	1.856 ± 0.391	2.622 ± 0.416	4.200 ± 0.376
HuH7‐PCDH	1.168 ± 0.174	1.638 ± 0.146	2.286 ± 0.040	2.858 ± 0.241	4.760 ± 0.051
HuH7‐PCDH‐CRKL	1.262 ± 0.106	2.578 ± 0.036	4.286 ± 0.307	5.888 ± 0.241	9.292 ± 0.162

### 3.3 CRKL promotes glycogen synthesis of hepatocarcinoma cells

**FIGURE 4:**
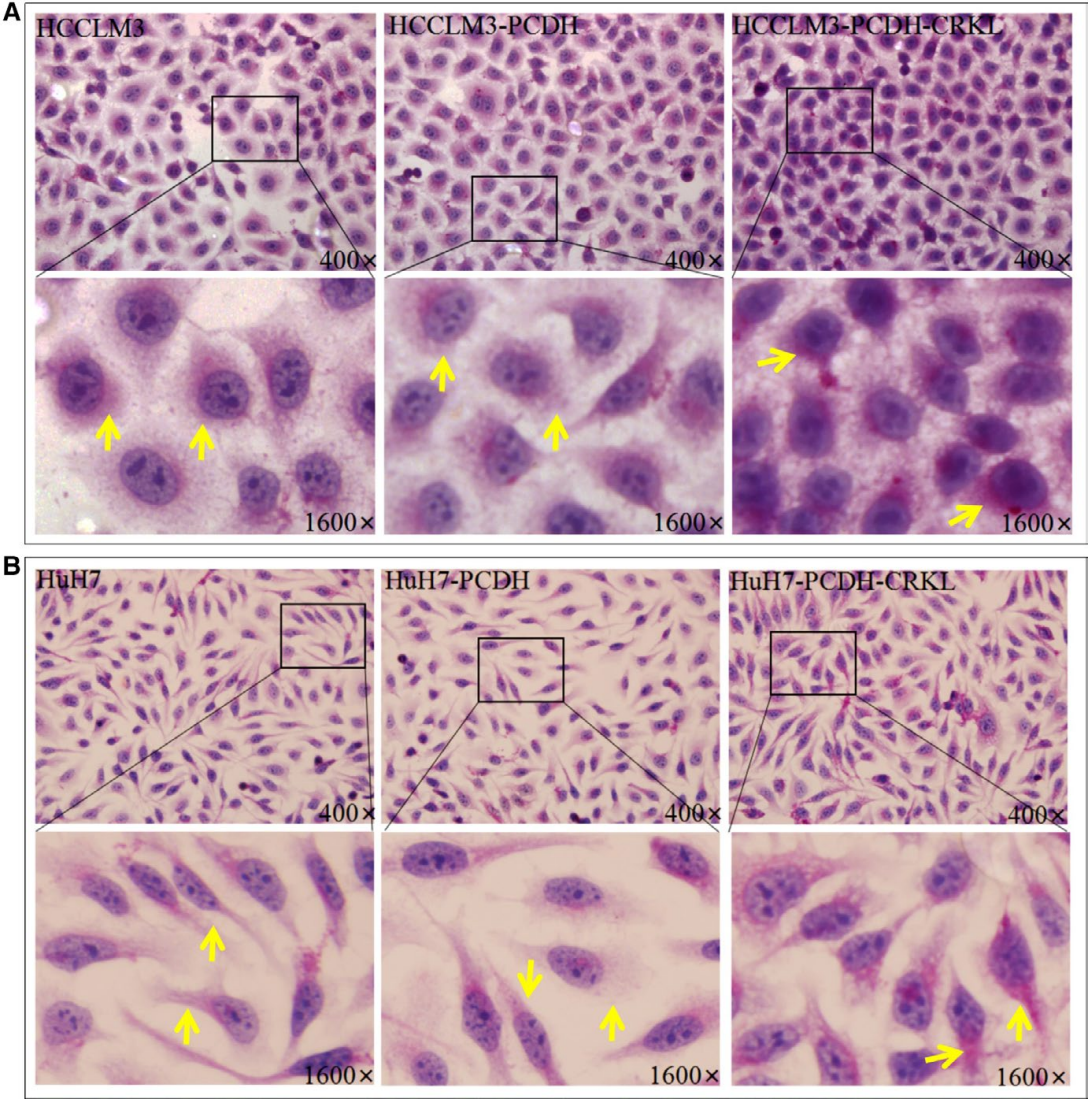
The influence of CRKL overexpression on the glycogen syntheses of HCCLM3 (A) and HuH7 (B) cells by PAS assays

Glycogen synthesis is another important indicator of glucose metabolism. PAS assay showed CRKL overexpression promoted glycogen synthesis of hepatocarcinoma cells (Figure 4). Compared to HCCLM3‐PCDH and HuH7‐PCDH, HCCLM3‐PCDH‐CRKL and HuH7‐PCDH‐CRKL cells exhibited strong positive PAS reaction with amaranth spherical particle glycogen was distributed in cytoplasm. The results further indicate that CRKL promotes glucose metabolism of hepatocarcinoma cells.

### 3.4 CRKL promotes glucose metabolism of hepatocarcinoma cells by activating the PI3K/AKT pathway

**FIGURE 5:**
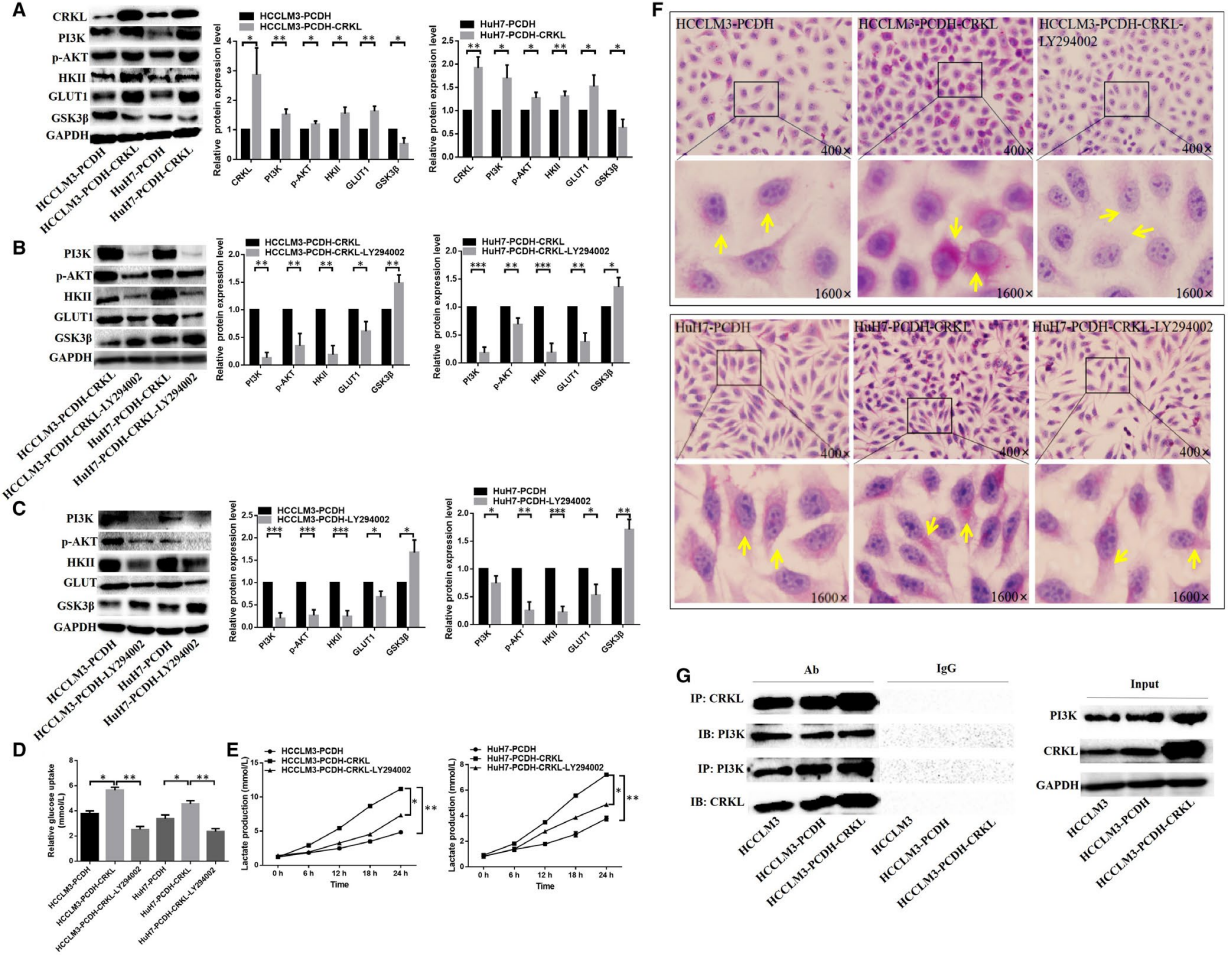
CRKL promotes glucose metabolism of hepatocarcinoma cells by activating the PI3K/AKT pathway. A, The influence of CRKL overexpression on protein expression levels of PI3K, p‐AKT, HKII, GLUT1 and GSK3β. The expression level changes of PI3K, p‐AKT, HKII, GLUT1 and GSK3β in HuH7‐PCDH‐CRKL and HCCLM3‐PCDH‐CRKL cells (B) or in HuH7‐PCDH and HCCLM3‐PCDH cells (C) after treatment with LY294002. D, The glucose uptake was decreased after blocking PI3K/AKT pathway in HuH7‐PCDH‐CRKL and HCCLM3‐PCDH‐CRKL cells. E, The lactate production was reduced after blocking PI3K/AKT pathway in HuH7‐PCDH‐CRKL and HCCLM3‐PCDH‐CRKL cells. F, The glycogen synthesis was weakened after blocking PI3K/AKT pathway in HuH7‐PCDH‐CRKL and HCCLM3‐PCDH‐CRKL cells. G, Co‐IP assay measured the interaction between CRKL and PI3K

The underlying molecular mechanism of CRKL in glucose metabolism is unclear. We found a remarkable positive relationship between the expression levels of CRKL and PI3K in HCC tissues and cells. Meanwhile, KEGG analysis revealed that CRKL deregulation is related to the PI3K/AKT pathway (Figure 1B). CRKL overexpression activated the PI3K/AKT pathway (Figure 5A). CRKL overexpression increased the protein expression levels of PI3K and p‐AKT by 52.3% (*P* = .0070), 18.3% (*P* = .0492) or 69.7% (*P* = .0134), 27.3% (*P* = .0170) in HCCLM3 or HuH7 cells. Moreover, the expression levels of glucose metabolism‐related molecules were also changed after CRKL overexpression (Figure 5A). CRKL overexpression increased the protein expression levels of HKII and GLUT1 by 55.3% (*P* = .0112) and 63.0% (*P* = .0034) and decreased the protein expression level of GSK3β by 47.0% (*P* = .014) in HCCLM3 cells; meanwhile, CRKL overexpression increased the protein expression levels of HKII and GLUT1 by 30.6% (*P* = .0086) and 52.0% (*P* = .0214) and decreased the protein expression level of GSK3β by 36.7% (*P* = .0243) in HuH7 cells. The results indicate that CRKL may promote glucose metabolism of hepatocarcinoma cells by activating the PI3K/AKT pathway.

The linkage of the PI3K/AKT pathway to CRKL‐mediated glucose metabolism was validated using LY294002. The treatment of HCCLM3‐PCDH‐CRKL and HuH7‐PCDH‐CRKL cells with LY294002 resulted in reduced protein expression of PI3K and p‐Akt, indicating LY294002 successfully blocked the PI3K/AKT pathway (Figure 5B). Meanwhile, the protein expression levels of HKII and GLUT1 were decreased, and the protein expression level of GSK3β was increased after blocking the PI3K/AKT pathway (Figure 5B,C). The results validate CRKL affects glucose metabolism of hepatocarcinoma cells by mediating the PI3K/AKT pathway.

Furthermore, we detected the glucose metabolism change after blocking the PI3K/AKT pathway. The Warburg effect was inhibited after blocking the PI3K/AKT pathway in HCCLM3‐PCDH‐CRKL and HuH7‐PCDH‐CRKL cells. The glucose uptake was decreased by 124.0% (*P* = .0043) and 92.3% (*P* = .0313) in HCCLM3‐PCDH‐CRKL and HuH7‐PCDH‐CRKL cells after blocking the PI3K/AKT pathway (Figure 5D, Table 3). Meanwhile, the lactate production was decreased by 34.3% (*P* = .0223), 39.9% (*P* = .0053), 47.6% (*P* = .0092), 34.5% (*P* = .0017) and 23.7% (*P* = .0383), 20.6% (*P* = .0464), 30.5% (*P* = .0132), 32.3% (*P* = .0140) in HCCLM3‐PCDH‐CRKL and HuH7‐PCDH‐CRKL cells at 6, 12, 18 and 24 hours after blocking the PI3K/AKT pathway (Figure 5E, Table 4). Moreover, the glycogen synthesis was weakened after blocking the PI3K/AKT pathway in HCCLM3‐PCDH‐CRKL and HuH7‐PCDH‐CRKL cells, the PAS‐positive reaction was weakened and the content of glycogen was obviously reduced after blocking the PI3K/AKT pathway (Figure 5F). These results indicate that CRKL regulates glucose metabolism of hepatocarcinoma cells by mediating PI3K/AKT pathway.

**TABLE 3:** The relative glucose uptake amounts of hepatocarcinoma cells

Cell	Glucose uptake (mmol/L)	Cell	Glucose uptake (mmol/L)
HCCLM3‐PCDH	3.778 ± 0.126	HuH7‐PCDH	3.383 ± 0.164
HCCLM3‐PCDH‐CRKL	5.640 ± 0.135	HuH7‐PCDH‐CRKL	4.552 ± 0.147
HCCLM3‐PCDH‐CRKL‐LY294002	2.516 ± 0.144	HuH7‐PCDH‐CRKL‐LY294002	2.368 ± 0.128

**TABLE 4:** The lactate production amounts of hepatocarcinoma cells

Cell	0 h (mmol/L)	6 h (mmol/L)	12 h (mmol/L)	18 h (mmol/L)	24 h (mmol/L)
HCCLM3‐PCDH	1.218 ± 0.060	1.830± 0.042	2.472 ± 0.057	3.502 ± 0.06675	5.012 ± 0.086
HCCLM3‐PCDH‐CRKL	1.204 ± 0.049	2.924 ± 0.083	5.420 ± 0.161	8.682 ± 0.314	11.200 ± 0.453
HCCLM3‐PCDH‐CRKL‐LY294002	1.424 ± 0.060	1.920 ± 0.043	3.258 ± 0.100	3.948 ± 0.116	6.332 ± 0.143
HuH7‐PCDH	0.830 ± 0.066	1.364 ± 0.064	1.784 ± 0.049	2.548 ± 0.089	3.778 ± 0.079
HuH7‐PCDH‐CRKL	0.918 ± 0.091	1.818 ± 0.087	3.490 ± 0.080	5.562 ± 0.088	7.202 ± 0.152
HuH7‐PCDH‐CRKL‐LY294002	0.814 ± 0.082	1.386 ± 0.053	2.768 ± 0.060	3.866 ± 0.067	4.874 ± 0.148

A previous study reported that the SH3N domain of CRKL could binding to the p85 PxxP motif of PI3K and resulted in activated PI3K.^46^ We performed a Co‐IP experiment to confirm the direct interaction between CRKL and PI3K in HCCLM3 cells. Our results obviously demonstrated that CRKL or PI3K protein band presented in the immunoprecipitated CRKL‐PI3K complexes with antibodies against PI3K or CRKL. Moreover, the amount of CRKL‐PI3K complex was more in HCCLM3‐PCDH‐CRKL cells than in HCCLM3‐PCDH cells (Figure 5G). Our results illuminate that CRKL directly binds to PI3K to positively mediate the expression of PI3K.

## 4 DISCUSSION

Metabolism involves the biological processes that allow healthy cells to maintain energy balance.^21^ Malignant cells reprogram their metabolism and energy production for their rapid proliferation and survival in severe environments due to the mutation of oncogenes and the inactivation of tumour suppressor genes.^22^ The dysregulated metabolism represents an adaptive advantage that facilitates growth and metastasis of tumour cell during tumorigenesis.^23^ Most cancer cells rely on the Warburg effect as a source of ATP. The change of tumour metabolism, particularly glucose metabolism, is regarded as a target for anti‐cancer therapy.^24^, ^25^ Better understanding of the molecular mechanism underlying energy metabolism is urgently needed in order to discover novel therapeutic targets and strategies to fight cancers.

CRKL deregulation is associated with various cancers, which is an interesting biomarker for diagnosis, therapy and prognosis of tumours.^13^ Previously, we found that CRKL was overexpressed in hepatocarcinoma patient tumour tissues and cells, indicating CRKL overexpression potentially e promoted hepatocarcinogenesis.^19^, ^20^ We also found that CRKL affected the migration and invasion potentials of HCCLM3, HuH7 and HepG2 cells.^19^, ^20^ Dysregulated metabolism of tumour cell provides an acidic microenvironment that facilitates tumour cell migration and invasion, CRKL deregulation associates with metastasis, and we speculate that CRKL may affect the energy metabolism of tumour cells. So, in the current study, we investigated the potential role of CRKL in energy metabolism of hepatocarcinoma.

Warburg effect is one of the best characterized metabolic disorders during cancer development and progression, which increases glucose uptake and lactate production.^5^ Glucose uptake and lactate production are important biological processes of energy metabolism.^10^ We measured the effect of CRKL overexpression on Warburg effect of hepatocarcinoma cells. We found that CRKL enhanced glucose uptake of HCCLM3 and Huh7 cells (Figure 3C, Table 1). Glucose is not able to across the plasma membrane on its own due to the hydrophilic composition of glucose; therefore, to overcome this condition, cancer cells induce GLUTs expression.^26^ GLUTs are transmembrane proteins responsible for facilitating the transport of extracellular glucose across the plasma membrane into cells during the glucose metabolism process.^27^, ^28^ GLUT1 is one of the most widely expressed isoforms in a variety of cells and has been reported to be up‐regulated in various cancers; it is mostly relevant to the glucose metabolism.^29^, ^30^ We found CRKL overexpression increased the protein expression level of GLUT1 in HCCLM3 and Huh7 cells (Figure 5A). Meanwhile, we found CRKL enhanced lactate production of HCCLM3 and Huh7 cells (Figure 3D,E, Table 2). The glycolytic pathway contains a series of 10 reactions, and the enzymes involved in the glycolysis are all potential targets for inhibitors used in anti‐cancer therapy.^31^ During the first step of glycolysis, glucose is transformed into glucose‐6 phosphate through the phosphorylation of the 6‐hydroxyl group of glucose by the enzyme hexokinase (HK).^32^, ^33^ The HK family contains four isoforms: I, II, III and IV. HKII is frequently overexpressed in a variety of cancers and is the major enzyme which is closely involved in glycolysis.^34^, ^35^, ^36^ We found that CRKL overexpression increased the protein expression level of HKII in HCCLM3 and Huh7 cells (Figure 5A). Taken together, our findings strongly support the notion that CRKL enhances the Warburg effect of hepatocarcinoma cells.

Liver is a crucial organ in glycogen synthesis, glucose metabolism and blood glucose maintenance.^37^ Glycogen is thought to be ‘a store of glucose’.^38^ Hepatic glycogen synthesis plays a crucial role in maintaining normal glucose homeostasis.^39^ We found CRKL overexpression promoted glycogen synthesis of HCCLM3 and HuH7 cells (Figure 4). Glycogen synthase kinase 3 (GSK3) is the primary regulatory kinase for glycogen synthase (GS) through phosphorylating and inactivating GS, including GSK3α and GSK3β,^40^, ^41^ GSK3β plays an important role in the mediation of blood glucose homeostasis.^42^, ^43^ We also found that CRKL overexpression increased the protein expression level of GSK3β in HCCLM3 and Huh7 cells (Figure 5A). Our findings strongly support the notion that CRKL enhances the glycogen synthesis of hepatocarcinoma cells.

The PI3K/Akt pathway plays a crucial role in the mediation of glucose metabolism through its downstream effector molecules, including promotion of glycolysis, glucose uptake, glycogen synthesis and inhibition of gluconeogenesis in the liver.^44^ Akt increases the glycolysis rate by promoting transcription and plasma membrane localization of GLUT1.^45^ Meanwhile, AKT can enhance HKII activity to promote glycolysis, at least in part, by increasing its association with a voltage‐dependent anion channel at the outer mitochondrial membrane.^46^ In addition, AKT can directly phosphorylate GSK3β, thereby rendering the kinase inactive and thus promoting glycogen synthesis.^47^ Previously, our proteomic results showed that CRKL deregulation related to the PI3K/Akt pathway (Figure 1B), PI3K was up‐regulated in HCC tissues (Figure 2A) and cells (Figure 2B). Meanwhile, CRKL up‐regulation was positively correlated with PI3K overexpression (Figure 2C). We speculate that CRKL may mediate glucose metabolism of hepatocarcinoma cells via PI3K/AKT pathway. In confirmation of the hypothesis, our results showed that CRKL overexpression enhanced PI3K and p‐AKT expression levels in HCCLM3 and Huh7 cells (Figure 5A). Furthermore, blocking the PI3K/AKT pathway resulted in decreased protein expression levels of GLUT1 and HKII, and increased protein expression level of GSK3β (Figure 5B,C). Meanwhile, the promotion effects of CRKL overexpression on the glucose uptake (Figure 5D), lactate production (Figure 5E) and glycogen synthesis (Figure 5F) of HCCLM3 and Huh7 cells were weakened. The SH3N domain of CRKLcan direct bind to the p85 PxxP motif of PI3K resulted in activated PI3K ^48^ for generating the phosphoinositide phosphates PIP2 and PIP3 at the inner surface of the plasma membrane. Then, Akt bound to PIP3 via PH domain, simultaneously being phosphorylated at Thr308 by phosphoinositide dependent kinase‐1 (PDK1) binding to PIP3, and phosphorylated its downstream target substrates to exhibit diverse biological functions.^49^ We have shown that CRKL directly binds to PI3K in HCCLM3 cells further indicating the direct interaction between CRKL and PI3K (Figure 5G). Our results indicate that CRKL regulates the glucose metabolism of hepatocarcinoma cells via the PI3K/AKT pathway.

Some compounds have been developed to interfere with the metabolic pathway by inhibiting metabolic enzymes which are important for tumour growth; however, normal cells also have the same metabolic requirements as cancer cells; therefore, one of the most effective anti‐cancer metabolism strategies is selectively and effectively inhibits metabolic enzymes but without harming the normal cells, molecule‐targeted therapy is an effective approach for treatment of tumour, and this therapeutic strategy by targeting proteins which are highly active or overexpressed in cancer cells would minimize the effect of anti‐cancer drugs in the normal cell. Understanding energy metabolism will give us an idea to develop new effective anti‐cancer therapies that target cancer energy production pathways.^6^ Many proteins involved in glycolysis are overexpressed in cancer cells but not all can be safely targeting without harming the normal cell. HKII is overexpressed in cancer cells, while its lowexpressed in normal cells, as HKII is specifically required by cancer cells, therefore inhibiting HKII can interfere with cancer progression, which provides a window to target HKII without harming normal cell.^50^, ^51^ We found CRKL promotes HCC hepatocarcinogenesis through enhancing cancer cells’ glucose metabolism via increasing GLUT1 expression, potentiating HKII activity and inactivating GSK3β activity, in the future, we hope the targeted‐CRKL drug will be designed to effectively inhibit glucose metabolism via increasing GLUT1 expression, potentiating HKII activity and inactivating GSK3β activity, which provides fundamental sight of molecule‐targeted therapy of cancer, molecule‐targeted therapy will represent the future development direction of treatment of tumours.

Cancer starvation therapy is emerging as an effective method for suppressing tumour growth and survival through blocking blood supply, depriving glucose/oxygen/nutrients supply.^52^ However, several undesirable properties of these agents, such as low targeting efficacy, undesired systemic side effects, elevated tumour hypoxia, induced drug resistance and increased tumour metastasis risk, limit their future applications.^53^ To overcome these challenges, combination therapy of cancer starvation with molecule‐targeted therapy will be an efficient way, which can maximize the therapeutic efficiency.

**FIGURE 6:**
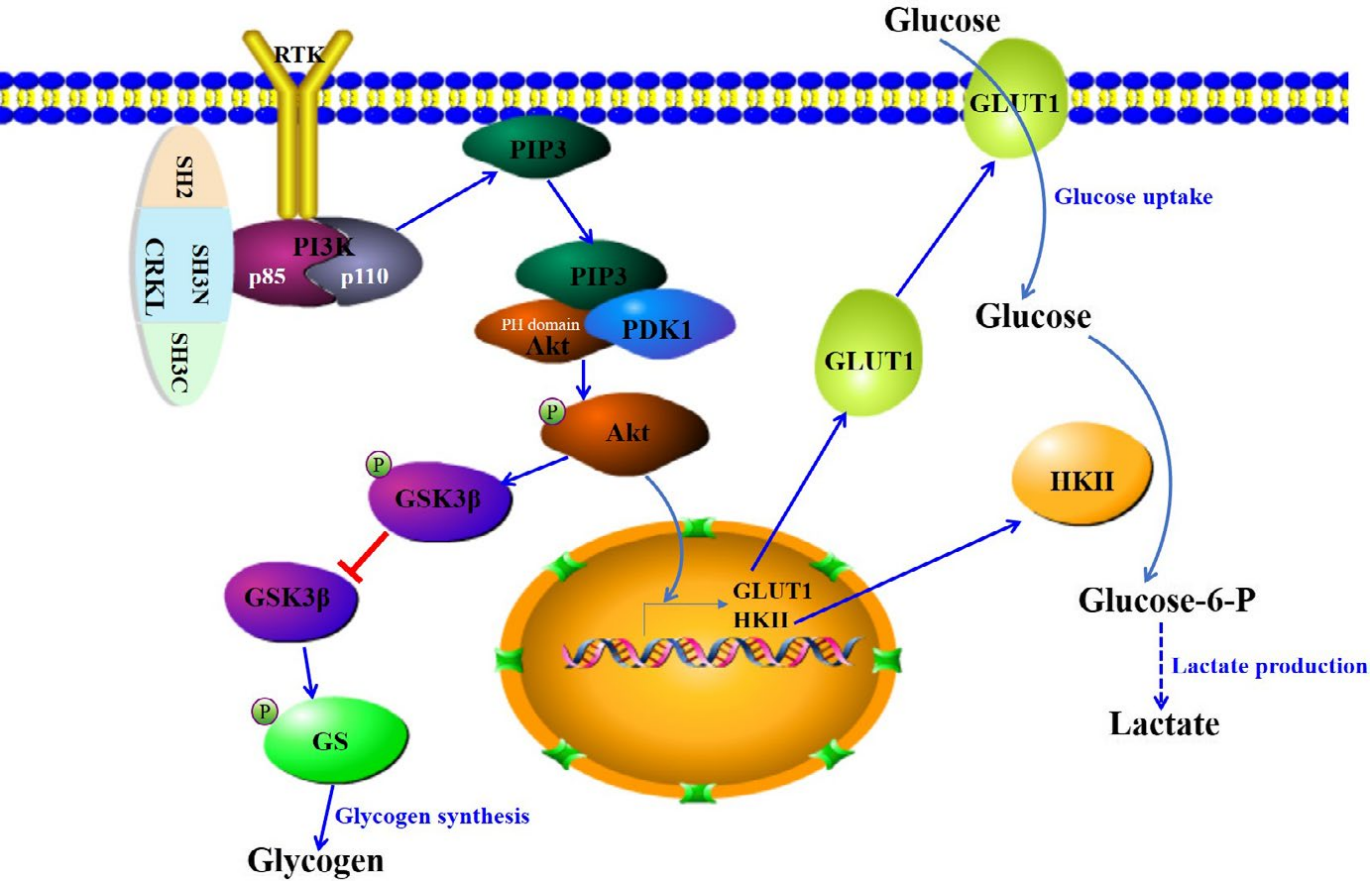
The schematic underlying mediation mechanism of CRKL on glucose metabolism in hepatocarcinoma. CRKL promotes glycolysis and glycogen synthesis through the PI3K/Akt pathway by increasing GLUT1 expression, potentiating HKII activity and inactivating GSK3β activity

As illustrated in Figure 6, the direct binding of SH3N domain of CRKL to the p85 PxxP motif of PI3K result in activated PI3K for generating the PIP2 and PIP3 at the inner membrane. Then, Akt binds to PIP3 via PH domain, simultaneously get phosphorylated at Thr308 by PDK1 binding to PIP3. Subsequently, p‐Akt promotes transcription and translocation of GLUT1 from the endomembrane to the cell surface; then, GLUT1 promotes the transport of extracellular glucose across the plasma membrane into the cells. In addition, p‐Akt promotes transcription and potentiates activity of HKII; then, glucose is transformed into glucose‐6‐P by HKII and undergoes glycolysis followed by the rapid conversion of pyruvate into lactate. Meanwhile, p‐AKT directly phosphorylates GSK3β lead to the inactivation of the kinase, the inactivated GSK3β is unable to phosphorylate and inactivate GS, subsequently glycogen is synthesized by GS.

Current work demonstrates CRKL‐PI3K/Akt regulatory pathway in glucose metabolism of hepatocarcinoma. CRKL up‐regulation potentially promotes hepatocarcinogenesis via enhancing cancer cells’ glucose metabolism through increasing GLUT1 expression, potentiating HKII activity and inactivating GSK3β activity. Herein, our work highlights the clue to suppress CRKL in inhibiting glucose metabolism of hepatocarcinoma cells, which provides a new fundamental sight of molecule‐targeted therapy of cancer.

## CONFLICT OF INTEREST

The authors declare that they have no competing interests.

## AUTHOR CONTRIBUTIONS

**Chunmei Guo:** Investigation (equal); Methodology (equal); Writing‐original draft (equal); Writing‐review & editing (equal). **Chao Gao:** Investigation (equal); Methodology (equal). **Xinxin Lv:** Investigation (equal). **Dongting Zhao:** Investigation (equal). **Frederick T. Greenaway:** Writing‐review & editing (equal). **Lihong Hao:** Data curation (equal); Software (equal). **Yuxiang Tian:** Data curation (equal); Software (equal); Validation (equal). **Shuqing Liu:** Funding acquisition (equal); Writing‐review & editing (equal). **Ming‐Zhong Sun:** Funding acquisition (equal); Writing‐review & editing (equal).

## ETHICAL APPROVAL AND CONSENT TO PARTICIPATE

This study has been conducted in accordance with ethical standards and has been approved by the authors’ institutional review board. The study protocol was approved by the Medical Ethics Committee of Dalian Medical University.

## Data Availability

The data support the findings of this study are available from the corresponding author upon reasonable request.

## References

[1] Siege RL , Miller KD , Jemal A . Cancer statistics. Cancer J Clin. 2019;69(1):7‐34. 10.3322/caac.21551 30620402

[2] El‐Serag HB , Rudolph KL . Hepatocarcinoma: epidemiology and molecular carcinogenesis. Gastroenterology. 2017;132(7):2557‐2576. 10.1053/j.gastro.2007.04.061 17570226

[3] Alqahtani A , Khan Z , Alloghbi A , Said Ahmed TS , Ashraf M , Hammouda DM . Hepatocarcinoma: molecular mechanisms and targeted therapies. Medicina. 2019;155(9):526. 10.3390/medicina55090526 PMC6780754 31450841

[4] Aravalli RN , Steer CJ , Cressman EN . Molecular mechanisms of hepatocarcinoma. Hepatology. 2008;48(6):2047‐2063. 19003900 10.1002/hep.22580

[5] Pavlova NN , Thompson CB . The emerging hallmarks of cancer metabolism. Cell Metab. 2016;23(1):27‐47. 26771115 10.1016/j.cmet.2015.12.006 PMC4715268

[6] Marbaniang C , Kma L . Dysregulation of glucose metabolism by oncogenes and tumor suppressors in cancer cells. Asian Pac J Cancer Prev. 2018;19(9):2377‐2390. 30255690 10.22034/APJCP.2018.19.9.2377 PMC6249467

[7] Laurent S , Claudiu TS , Khalid OA . The Warburg effect and the hallmarks of cancer. Anticancer Agents Med Chem. 2017;17(2):164‐170. 27804847 10.2174/1871520616666161031143301

[8] Fadaka A , Ajiboye B , Adeleke O , Adewale O , Olayide I , Emuowhochere R . Biology of glucose metabolization in cancer cells. J Toxicol Sci. 2017;3(2):45‐51.

[9] Lu JR . The Warburg metabolism fuels tumor metastasis. Cancer Metastasis Rev. 2019;38(1–2):157‐164. 30997670 10.1007/s10555-019-09794-5

[10] Lebelo MT , Joubert AM , Visagie MH . Warburg effect and its role in tumourigenesis. Arch Pharm Res. 2019;42(10):833‐847. 31473944 10.1007/s12272-019-01185-2

[11] Vander Heiden MG , Cantley LC , Thompson CB . Understanding the Warburg effect: the metabolic requirements of cell proliferation. Science. 2009;324(5930):1029‐1033. 19460998 10.1126/science.1160809 PMC2849637

[12] Birge RB , Kalodimos C , Inagaki F , Tanaka S . Crk and CRKL adaptor proteins: networks for physiological and pathological signaling. Cell Commun Signal. 2009;7(1):13. 19426560 10.1186/1478-811X-7-13 PMC2689226

[13] Guo CM , Liu SQ , Sun M‐Z . The role of CT10 regulation of kinase‐like in cancer. Future Oncol. 2014;10(16):2687‐2697. 25531052 10.2217/fon.14.199

[14] Lin QY , Sun M‐Z , Guo CM , Shi J , Chen X , Liu SQ . CRKL overexpression suppresses *in vitro* proliferation, invasion and migration of murine hepatocarcinoma Hca‐P cells. Biomed Pharmacother. 2015;69:11‐17. 25661331 10.1016/j.biopha.2014.10.025

[15] Park T , Koptyra M , Curran T . Fibroblast growth requires CT10 regulator of kinase (Crk) and Crk‐like (CrkL). J Biol Chem. 2016;291(51):26273‐26290. 27807028 10.1074/jbc.M116.764613 PMC5159491

[16] Emily SB , Morag P . Models of Crk adaptor proteins in cancer. Genes Cancer. 2012;3(5–6):341‐352. 23226572 10.1177/1947601912459951 PMC3513787

[17] Sriram G , Birge RB . Emerging roles for crk in human cancer. Genes Cancer. 2010;1(11):1132‐1139. 21779437 10.1177/1947601910397188 PMC3092275

[18] Shi J , Meng LL , Sun M‐Z , et al. CRKL knockdown promotes *in vitro* proliferation, migration and invasion, *in vivo* tumor malignancy and lymph node metastasis of murine hepatocarcinoma Hca‐P cells. Biomed Pharmacother. 2015;71(1):84‐90. 25960220 10.1016/j.biopha.2015.02.022

[19] Guo CM , Zhao DT , Zhang QL , Liu SQ , Sun M‐Z . miR‐429 suppresses tumor migration and invasion by targeting CRKL in hepatocellular carcinoma via inhibiting Raf/MEK/ERK. Sci Rep. 2018;8(1):2375. 29403024 10.1038/s41598-018-20258-8 PMC5799248

[20] Guo CM , Gao C , Zhao DT , et al. A novel ETV6‐miR‐429‐CRKL regulatory circuitry contributes to aggressiveness of hepatocellular carcinoma. J Exp Clin Cancer Res. 2020;39(1):70. 32326970 10.1186/s13046-020-01559-1 PMC7178969

[21] Karolien V , Geert‐Jan G , Liesbet M , et al. The metabolic landscape of lung cancer: new insights in a disturbed glucose metabolism. Front Oncol. 2019;9:1215. 31803611 10.3389/fonc.2019.01215 PMC6873590

[22] Levine AJ , Puzio‐Kuter AM . The control of the metabolic switch in cancers by oncogenes and tumor suppressor genes. Sciences. 2010;330(6009):1340‐1344. 10.1126/science.1193494 21127244

[23] Pedroza‐Torres A , Romero‐Córdoba SL , Justo‐Garrido M , et al. MicroRNAs in tumor cell metabolism: roles and therapeutic opportunities. Front Oncol. 2019;9:1404. 31921661 10.3389/fonc.2019.01404 PMC6917641

[24] Lu W , Cao FH , Wang SJ , Sheng XM , Ma J . LncRNAs: the regulator of glucose and lipid metabolism in tumor cells. Front Oncol. 2019;9:1099. 31850189 10.3389/fonc.2019.01099 PMC6901916

[25] Sousa B , Pereira J , Paredes J . The crosstalk between cell adhesion and cancer metabolism. Int J Mol Sci. 2019;20(8):1933. 10.3390/ijms20081933 PMC6515343 31010154

[26] Meng Y , Xu X , Luan H , et al. The progress and development of GLUT1 inhibitors targeting cancer energy metabolism. Future Med Chem. 2019;11(17):2333‐2352. 31581916 10.4155/fmc-2019-0052

[27] Bonatelli M , Silva ECA , Cárcano FM , et al. The Warburg effect is associated with tumor aggressiveness in testicular germ cell tumors. Front Endocrinol. 2019;10:417. 10.3389/fendo.2019.00417 PMC6610306 31316469

[28] Galochkina T , Chong MNF , Challali L , Abbar S , Etchebest C . New insights into GluT1 mechanics during glucose transfer. Sci Rep. 2019;9(1):998. 30700737 10.1038/s41598-018-37367-z PMC6353926

[29] Xiao HJ , Wang J , Yan WX , et al. GLUT1 regulates cell glycolysis and proliferation in prostate cancer. Prostate. 2018;78(2):86‐94. 29105798 10.1002/pros.23448

[30] Schmidt M , Voelker HU , Kapp M , Krockenberger M , Dietl J , Kammerer U . Glycolytic phenotype in breast cancer: activation of Akt, up‐regulation of GLUT1, TKTL1 and down‐regulation of M2PK. J Cancer Res Clin Oncol. 2010;136(2):219‐225. 19655166 10.1007/s00432-009-0652-y PMC11828084

[31] Mathupala SP , Ko YH , Pedersen PL . Hexokinase‐2 bound to mitochondria: cancer's stygian link to the "Warburg Effect" and a pivotal target for effective therapy. Semin Cancer Biol. 2009;19(1):17‐24. 19101634 10.1016/j.semcancer.2008.11.006 PMC2714668

[32] Roberts DJ , Miyamoto S . Hexokinase II integrates energy metabolism and cellular protection: Akting on mitochondria and TORCing to autophagy. Cell Death Differ. 2015;22(2):248‐257. 25323588 10.1038/cdd.2014.173 PMC4291497

[33] Luo FX , Li Y , Yuan F , Zuo JL . Hexokinase II promotes the Warburg effect by phosphorylating alpha subunit of pyruvate dehydrogenase. Chin J Cancer Res. 2019;31(3):521‐532. 31354221 10.21147/j.issn.1000-9604.2019.03.14 PMC6613503

[34] Yao J , Liu J , Zhao W . By blocking hexokinase‐2 phosphorylation, limonin suppresses tumor glycolysis and induces cell apoptosis in hepatocellular carcinoma. Onco Targets Ther. 2018;11:3793‐3803. 30013360 10.2147/OTT.S165220 PMC6037266

[35] Guo YJ , Wei LB , Zhou YX , et al. Flavonoid GL‐V9 induces apoptosis and inhibits glycolysis of breast cancer via disrupting GSK‐3β‐modulated mitochondrial binding of HKII. Free Radic Biol Med. 2020;146:119‐129. 31669347 10.1016/j.freeradbiomed.2019.10.413

[36] Li RL , Yang WD . Gomisin J inhibits the glioma progression by inducing apoptosis and reducing HKII‐regulated glycolysis. Biochem Biophys Res Commun. 2020;529(1):15‐22. 32560813 10.1016/j.bbrc.2020.05.109

[37] Petersen MC , Vatner DF , Shulman GI . Regulation of hepatic glucose metabolism in health and disease. Nat Rev Endocrinol. 2017;13(10):572‐587. 28731034 10.1038/nrendo.2017.80 PMC5777172

[38] Radziuk J , Pye S . Hepatic glucose uptake, gluconeogenesis and the regulation of glycogen synthesis. Diabetes Metab Res Rev. 2001;17(4):250‐270. 11544610 10.1002/dmrr.217

[39] Nozaki Y , Max P , Zhang DY , et al. Metabolic control analysis of hepatic glycogen synthesis *in vivo* . Proc Natl Acad Sci. 2020;117(14):8166‐8176. 32188779 10.1073/pnas.1921694117 PMC7149488

[40] Frame S , Cohen P . GSK3 takes centre stage more than 20 years after its discovery. Biochem J. 2001;359(1):1‐16. 11563964 10.1042/0264-6021:3590001 PMC1222116

[41] Rayasam GV , Tulasi VK , Sodhi R , Davis JA , Ray A . Glycogen synthase kinase 3: more than a namesake. Br J Pharmacol. 2009;156(6):885‐898. 19366350 10.1111/j.1476-5381.2008.00085.x PMC2697722

[42] Martin SA , Souder DC , Miller KN , et al. GSK3β regulates brain energy metabolism. Cell Rep. 2018;23(7):1922‐1931. 29768193 10.1016/j.celrep.2018.04.045 PMC6082412

[43] Wang KP , Wang HX , Liu YG , et al. Dendrobiurn officinale polysaccharide attenuates type 2 diabetes mellitus via the regulation of PI3K/Akt‐mediated glycogen synthesis and glucose metabolism. J Funct Foods. 2018;40:261‐271.

[44] Hoxhaj G , Manning BD . The PI3K‐AKT network at the interface of oncogenic signalling and cancer metabolism. Nat Rev Cancer. 2020;20(2):74‐88. 31686003 10.1038/s41568-019-0216-7 PMC7314312

[45] Brooks RR , Nissim H . Is Akt the "Warburg kinase"? ‐Akt‐energy metabolism interactions and oncogenesis. Semin Cancer Biol. 2009;19(1):25‐31. 19130886 10.1016/j.semcancer.2008.11.010 PMC2814453

[46] Roberts DJ , Tan‐Sah VP , Smith JM , Miyamoto S . Akt phosphorylates HK‐II at Thr‐473 and increases mitochondrial HK‐II association to protect cardiomyocytes. J Biol Chem. 2013;288(33):23798‐23806. 23836898 10.1074/jbc.M113.482026 PMC3745326

[47] Chen H , Fajol A , Hoene M , et al. PI3K‐resistant GSK3 controls adiponectin formation and protects from metabolic syndrome. Proc Natl Acad Sci. 2016;113(20):5754‐5759. 27140617 10.1073/pnas.1601355113 PMC4878493

[48] Ylsmki L , Schmotz C , Ylsmki E , Saksela K . Reorganization of the host cell Crk(L)‐PI3 kinase signaling complex by the influenza A virus NS1 protein. Virology. 2015;484:146‐152. 26099693 10.1016/j.virol.2015.06.009

[49] Rai SN , Dilnashin H , Birla H , et al. The role of PI3K/Akt and ERK in neurodegenerative disorders. Neurotox Res. 2019;35(3):775‐795. 30707354 10.1007/s12640-019-0003-y

[50] Pastorino JG , Shulga N , Hoek JB . Mitochondrial binding of hexokinase II inhibits Bax‐induced cytochrome c release and apoptosis. J Biol Chem. 2002;277:7610‐7618. 11751859 10.1074/jbc.M109950200

[51] Hay N . Reprogramming glucose metabolism in cancer: can it be exploited for cancer therapy? Nat Rev Cancer. 2016;16:635‐649. 27634447 10.1038/nrc.2016.77 PMC5516800

[52] Selwan EM , Finicle BT , Kim SM , Edinger AL . Attacking the supply wagons to starve cancer cells to death. FEBS Lett. 2016;590:885‐907. 26938658 10.1002/1873-3468.12121 PMC4833639

[53] Yu SJ , Chen ZW , Zeng X , Chen XS , Gu Z . Advances in nanomedicine for cancer starvation therapy. Theranostics. 2019;9(26):8026‐8047. 31754379 10.7150/thno.38261 PMC6857045

